# Reproducibly sampling SARS-CoV-2 genomes across time, geography, and viral diversity

**DOI:** 10.12688/f1000research.24751.2

**Published:** 2020-10-28

**Authors:** Evan Bolyen, Matthew R. Dillon, Nicholas A. Bokulich, Jason T. Ladner, Brendan B. Larsen, Crystal M. Hepp, Darrin Lemmer, Jason W. Sahl, Andrew Sanchez, Chris Holdgraf, Chris Sewell, Aakash G. Choudhury, John Stachurski, Matthew McKay, Anthony Simard, David M. Engelthaler, Michael Worobey, Paul Keim, J. Gregory Caporaso

**Affiliations:** 1 Center for Applied Microbiome Science, Pathogen and Microbiome Institute, Northern Arizona University, Flagstaff, AZ, USA; 2 School of Informatics, Computing, and Cyber Systems, Northern Arizona University, Flagstaff, AZ, USA; 3 Laboratory of Food Systems Biotechnology, Institute of Food, Nutrition and Health, ETH Zurich, Switzerland; 4 Pathogen and Microbiome Institute, Northern Arizona University, Flagstaff, AZ, USA; 5 Department of Ecology and Evolutionary Biology, University of Arizona, Tucson, AZ, USA; 6 Pathogen and Microbiome Division, Translational Genomics Research Institute, Flagstaff, AZ, USA; 7 Department of Biological Sciences, Northern Arizona University, Flagstaff, AZ, USA; 8 Department of Statistics, University of California at Berkeley, Berkeley, CA, USA; 9 Theory and Simulation of Materials, École Polytechnique Fédérale de Lausanne, Lausanne, Switzerland; 10 Research School of Economics, Australian National University, ACT, Australia

**Keywords:** SARS-CoV-2, genome-sampler, QIIME 2, bioinformatics, genomics

## Abstract

The COVID-19 pandemic has led to a rapid accumulation of SARS-CoV-2 genomes, enabling genomic epidemiology on local and global scales. Collections of genomes from resources such as GISAID must be subsampled to enable computationally feasible phylogenetic and other analyses. We present genome-sampler, a software package that supports sampling collections of viral genomes across multiple axes including time of genome isolation, location of genome isolation, and viral diversity. The software is modular in design so that these or future sampling approaches can be applied independently and combined (or replaced with a random sampling approach) to facilitate custom workflows and benchmarking. genome-sampler is written as a QIIME 2 plugin, ensuring that its application is fully reproducible through QIIME 2’s unique retrospective data provenance tracking system. genome-sampler can be installed in a conda environment on macOS or Linux systems. A complete default pipeline is available through a Snakemake workflow, so subsampling can be achieved using a single command. genome-sampler is open source, free for all to use, and available at https://caporasolab.us/genome-sampler. We hope that this will facilitate SARS-CoV-2 research and support evaluation of viral genome sampling approaches for genomic epidemiology.

## Introduction

The intersection of the SARS-CoV-2 outbreak and the genomics revolution has led to the rapid accumulation of viral genomes that are fueling our epidemiological understanding of the global pandemic. However, the rate of genome sequencing is challenging our ability to conduct comprehensive analyses in a timely manner. Local networks of health care professionals, laboratory professionals, and researchers are rapidly generating genome sequences at an unprecedented rate and feeding these data into global community resources, such as GISAID ^1^ and GenBank ^2^. Contextualizing locally-derived genome sequences with those from global resources (e.g., as recently performed by the Arizona COVID-19 Genomics Union ^3^) enables phylogenetic analyses that can provide information about the relative roles of local transmission versus repeated introductions. This can help to evaluate the utility of control measures, such as stay-at-home orders. These sequencing data thus enable a new paradigm in epidemiology, which must be facilitated by computational workflows designed to handle this scale of data.

Contextualization of locally derived genome sequences will generally begin with two collections of sequences: those obtained from a global community resource and those obtained locally. The widely used NextStrain ^4^ platform refers to these sequence collections in their documentation as the *context sequences* and the *focal sequences*, respectively, and we adopt that terminology here.

To enable phylogenetic analysis of full-length SARS-CoV-2 genomes, for example with Bayesian methods or maximum likelihood methods with bootstrap support, subsampling the context sequences is essential for computational feasibility. To avoid introducing post-sequencing sampling biases into our analysis, we subsampled the context sequences across three axes: time, space (i.e., geographic dispersion of near neighbors of focal sequences), and viral genome diversity. Sampling across time is a prerequisite to reliable inference of molecular clock signal from the data by ensuring that our sample of viral genomes span as much time as possible and include the oldest available genomes. Sampling the context sequences to include near neighbors of the focal sequences that come from different geographic regions enables us to avoid erroneously describing groups of focal sequences as monophyletic. Sampling across viral diversity enables us to represent the known diversity of the virus in our analysis. Each of these steps additionally reduces the chance of over-represented genomes dominating the analysis. When data sets are relatively small, this process can be performed manually, but when numbers of context genomes measure in the thousands, tens of thousands, or even hundreds of thousands (which may be likely as the pandemic progresses), an automated and reproducible subsampling approach is essential to maximize efficiency and to avoid human error.

Here we present genome-sampler ^5^, a QIIME 2 plugin that enables other research teams to apply our context sequence subsampling workflow. Our subsampling workflow is compatible with tools such as NextStrain ^4^, which includes a similar but not identical subsampling process (details provided in the *Discussion* section). We believe that our workflow can reduce sampling bias in analysis of SARS-CoV-2 genomes, and could be applied for regionally focused analyses, such as ours, or globally focused analyses. QIIME 2 ^6^ ( https://qiime2.org) is a plugin-based bioinformatics software platform developed for microbiome multi-omics analysis. It includes a unique retrospective data provenance tracking system that ensures reproducibility of bioinformatics steps by recording details of all analysis steps (commands called, parameters and input arguments provided, as well as details of the computational environment where the analysis was run, such as versions of underlying software dependencies; see examples at https://view.qiime2.org and in Figure 2 of the QIIME 2 paper ^6^). We built this functionality as a QIIME 2 plugin because, given the pace at which SARS-CoV-2 genomics research is currently being carried out, human error in bioinformatics workflows is likely and the detailed record keeping needed to ensure reproducibility may be inadvertently skipped. QIIME 2 ensures that workflow errors could be detected retroactively and that workflows can be reproduced, even if detailed records are not kept while they are being run.

## Methods

### Implementation

genome-sampler ^5^ operates on three input files: a fasta file containing the unaligned context sequences, a fasta file containing the unaligned focal sequences, and a tab-separated text file containing metadata for the context sequences. The context sequences and metadata will typically be obtained by the user from a public repository such as GISAID. The focal sequences will typically be sequences that the team has compiled independently, for example from their locale.

### Operation

genome-sampler can be installed in a conda environment on macOS or Linux systems, as described in its installation documentation linked from the project website. The complete workflow can be applied in one step using the included Snakemake ^7^ workflow, or the steps can be applied individually.

**Figure 1.**
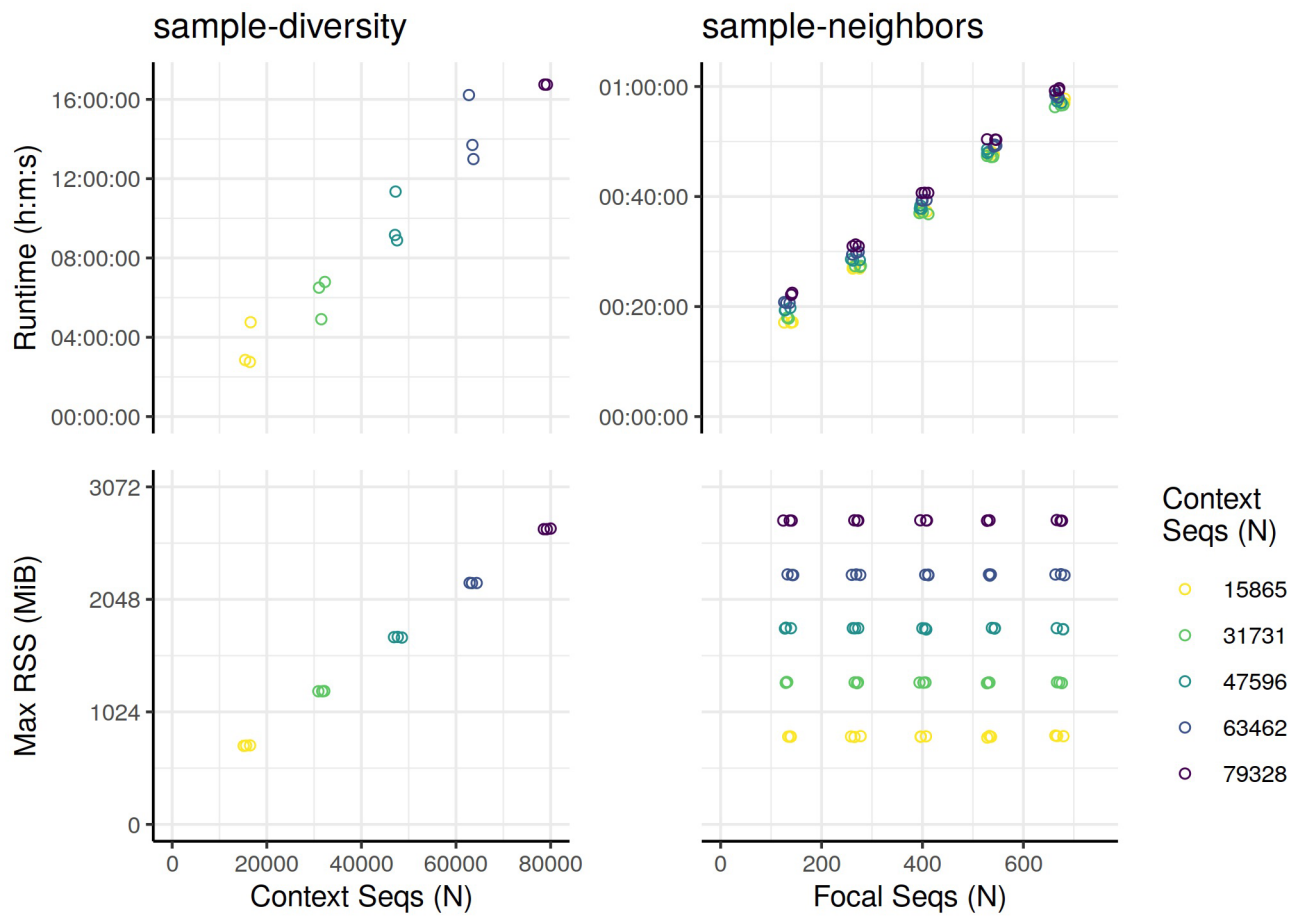
The runtime and memory requirements (top and bottom row, respectively) for sample-diversity and sample-neighbors (left and right column, respectively) are shown. Data was sourced from GISAID. Context sequences were resampled three times at each of the evenly spaced dataset sizes shown in the legend. The largest size (N=79,328) shows less variability because each subsample represented the entire dataset. The points are jittered on the x-axis to improve legibility, the y-axis remains un-jittered. *Max RSS* refers to *Memory (max resident set size)*. All benchmarks were run with 28 threads (details in *Operation* section).

Most steps in genome-sampler run very quickly (within a few seconds to a few minutes), however two steps ( sample-diversity and sample-neighbors) are much slower and highly dependent on dataset size and characteristics ( Figure 1). A benchmark was performed on a single node of the monsoon cluster computer at Northern Arizona University with an Intel(R) Xeon(R) Gold 6132 CPU (28 logical processors) and 196 GB of RAM. Both sample-neighbors and sample-diversity were run with 28 threads. Context sequences were resampled randomly three times for each dataset size. The runtime of sample-diversity scaled with the number of sequences provided in a linear fashion, with variability related primarily to the specific characteristics of each initial random subsample. For sample-neighbors, the number of context sequences had a limited impact on the runtime, and was instead more directly related to the number of focal sequences. Memory (max resident set size) grew linearly with the number of context sequences for both steps.

## Use case

**Figure 2.**
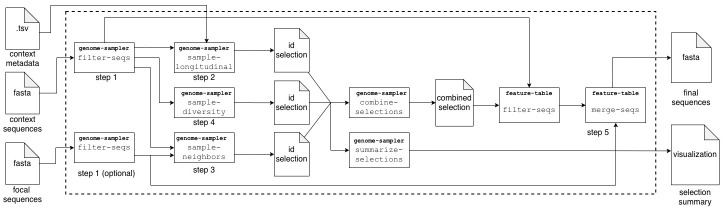
The genome-sampler workflow. This workflow samples context sequences for downstream phylogenetic analysis. Specific steps are represented by boxes: the QIIME 2 plugin name is bolded, and the action name in monospace font. Inputs and outputs are represented by folded-page file icons. The surrounding dashed box represents the Snakemake workflow which automates execution of the contained steps. Given context metadata, context sequences, and focal sequences, the Snakemake workflow will produce a fasta file which is ready for alignment and a summary of the sampling procedure as a QIIME 2 visualization.

Here we describe the series of steps taken by the genome-sampler ^5^ workflow (see Figure 2). In each step, any parameter values that can be overridden by the user are bolded. This description is accompanied by an online tutorial, available from the project website, which illustrates a use case focused on a small set of sequences obtained from GISAID. The tutorial is tested with each release of genome-sampler to ensure that all commands remain up to date.

The genome-sampler workflow works as follows:

1. Clean up and filter the context sequences.
  i. Filter sequences that contain non-IUPAC characters ^8^ as these characters can be problematic for downstream tools, such as sequence aligners or alignment viewers.
  ii. Remove any gap (“-” or “.”) characters, as this workflow is intended to work on unaligned sequences. (Aligned reference sequences can be provided as input since they will be unaligned in this step.)
  iii. Filter sequences that are composed of > **10%** N characters.
  iv. Optionally filter sequences with length less than a user-specified minimum length or greater than a user-specified maximum length.
2. Uniformly sample context sequences across time, selecting **7** sequences from each **7**-day period between the **earliest** and latest dates represented in the data set. If there are fewer than 7 sequences in any 7-day period, all sequences from that period are included in the result. These sequences are referred to as the *temporally sampled context sequences*. The user can optionally supply a start date, in which case any genomes from before that time will be excluded. **
3. Search focal sequences against context sequences to identify the **10** closest matches to each focal sequence. This is achieved using vsearch’s usearch_global option ^9^ at **99.99** percent identity. The resulting collections of closest matches are sampled to select **3** geographically distinct context sequences for each focal sequence for inclusion in the subsampled context sequence collection. This sampling procedure is weighted such that each geographic region has an equal probability of selection instead of each genome. This weighting prevents overrepresented regions from dominating the sample. This step ensures that any monophylies of target sequences are not artifacts of our sequence sampling approach. These sequences are referred to as the *geographically sampled context sequences*. (This step is achieved using sequence metadata, and can be parameterized so that this can be applied over any categorical metadata, not just geography.) **
4. Cluster the complete context sequence collection with vsearch’s cluster_fast option at **99.90** percent identity. The resulting cluster centroid sequences represent a divergent collection of the SARS-CoV-2 genomes and are referred to as the *diversity sampled context sequences*.
5. Combine the temporally, geographically, and diversity sampled context sequences with the focal sequence collection. The resulting collection of sequences will be deduplicated by sequence identifier, so sequences contained in multiple different subsamples are represented only once in the final sequence collection. This final collection of sequences should be used for downstream analysis.

## Discussion

### Resemblance to NextStrain context sequence sampling workflow

The NextStrain workflow also subsamples context sequences for its phylogenetic tree builds using augur ( https://github.com/nextstrain/augur) and scripts in their ncov repository ( https://github.com/nextstrain/ncov). Their workflow subsamples the context sequences across two axes: time and geography, prioritizing similarity to focal sequences when selecting sequences from different geographic regions. They sample across time by including a specified number of sequences per month for different regions. When determining the closest matches, percent identity is computed based on a multiple sequence alignment of all sequences, which is computed by aligning each sequence against a reference alignment using mafft ^10^.

Step 2 of our workflow is similar to their time sampling approach, but is independent of other variables such as geography. The workflows diverge more in Step 3, where we begin by identifying near neighbors of all focal sequences using global alignment search with vsearch. We then optionally sample across the geographic source of those sequences such that each geographic region represented in each collection of near neighbors has an equal probability of selection. We follow this with Step 4, where we sample the full genetic diversity of the context sequences by clustering them all against one another and including the resulting cluster centroid sequences in our final sequence collection. As far as we are aware, there is not an analog to our Step 4 in the NextStrain workflow.

Our workflow is modular in design to facilitate benchmarking and optimization of this essential context sequence sampling step. Our three sampling steps can be used individually or in any combination, and can be replaced with a random sampling step (the sample-random action) to allow evaluation of the importance of each step. At this stage, we do not claim that our workflow is better than the one used by NextStrain. We hope the similarity of our interfaces (both of which require the same input and output, are accessible through Snakemake, and use the same terminology to describe data) will allow for independent comparison of these and other approaches. In our next stage of work on this project, we plan to evaluate the impact of each subsampling step and their associated parameters on downstream phylogenetic results.

### Retrospective data provenance tracking system

The retrospective data provenance tracking system implemented in QIIME 2 differs from other systems such as Snakemake ^7^ or Galaxy ^11^, which we view as providing prospective data provenance tracking. For example, when a Snakemake file is used to run a workflow, that workflow is documented for reproducibility by the Snakemake file. However, if a user were to run the underlying commands independently, they must keep detailed records of their commands to ensure reproducibility of the analysis. This is not necessary with QIIME 2’s retrospective data provenance tracking system, which records steps regardless of whether the workflow is run using a tool like Snakemake or Galaxy, or whether individual components are run independently. Additionally, QIIME 2’s system assigns universally unique identifiers (UUIDs) to all execution steps, inputs, and outputs, so data can be unambiguously linked to workflow descriptions. QIIME 2 is therefore fully compatible with workflow engines such as Snakemake or Galaxy, but provides additional information which further ensures reproducibility.

We present genome-sampler ^5^, a QIIME 2 plugin that supports subsampling of genomic sequence collections based on time of genome isolation, geography of genome isolation, and genomic diversity, thus facilitating genomic epidemiology based on large numbers of genomes while reducing the possibility of post-sequencing sampling bias impacting conclusions. As the number of available SARS-CoV-2 genomes continues to increase rapidly, approaches such as this will be required to enable phylogenetic and other analyses of genome data.

## Data availability

### Source data

The context sequences and metadata used in the genome-sampler *Use case* were obtained from GISAID. Those genomes were sampled from patients in Arizona, USA, and published to GISAID by the Arizona COVID-19 Genomics Union (ACGU). The focal sequences and metadata used in the genome-sampler *Use case* were sequenced at a later time than the context sequences, also from patients in Arizona. The focal sequences were generated and assembled by the ACGU and are currently being added to GISAID. These context and focal sequences and associated metadata are all available for download for use in learning genome-sampler (see the project website). For analysis purposes, we recommend obtaining sequences from a public repository, such as GISAID or GenBank, as those sequences will be updated (for example to improve genome assemblies) before our tutorial data is updated.

## Software availability

**genome-sampler source code available at:** at https://github.com/caporaso-lab/genome-sampler.

**Archived source code and tutorial data at time of publication:** https://doi.org/10.5281/zenodo.3891818 ^5^.

**License:** BSD 3-Clause "New" or "Revised" License.

Documentation, written using Myst ( https://myst-parser.readthedocs.io/en/latest/) and rendered using Jupyter Book ( https://jupyterbook.org/), is available at http://caporasolab.us/genome-sampler/. If you need technical support, please post a question to the QIIME 2 Forum at https://forum.qiime2.org. We are very interested in contributions to genome-sampler from the community - please get in touch via the GitHub issue tracker or the QIIME 2 Forum if you’re interested in contributing.
