# Supplementary material for: Reproducibly sampling SARS-CoV-2 genomes across time, geography, and viral diversity
Source: F1000Res. 2020 Oct 28;9:657. Originally published 2020 Jun 29. [Version 2] doi: 10.12688/f1000research.24751.2 (PMC7814287; doi:10.12688/f1000research.24751.2)
Supplement: Peer review history

# Peer review: Reviewer response for version 2

**DOI:** 10.5256/f1000research.30305.r73795

**Published:** 2021-01-18

**Authors:** C. Titus Brown

**Affiliations:** 1 Department of Population Health and Reproduction, University of California, Davis, Davis, CA, USA

Thank you!

Are the conclusions about the tool and its performance adequately supported by the findings presented in the article?

Yes

Is the rationale for developing the new software tool clearly explained?

Yes

Is the description of the software tool technically sound?

Partly

Are sufficient details of the code, methods and analysis (if applicable) provided to allow replication of the software development and its use by others?

Partly

Is sufficient information provided to allow interpretation of the expected output datasets and any results generated using the tool?

Yes

Reviewer Expertise:

bioinformatics and software development

I confirm that I have read this submission and believe that I have an appropriate level of expertise to confirm that it is of an acceptable scientific standard.

# Peer review: Reviewer response for version 1

**DOI:** 10.5256/f1000research.27305.r67936

**Published:** 2020-08-27

**Authors:** C. Titus Brown

**Affiliations:** 1 Department of Population Health and Reproduction, University of California, Davis, Davis, CA, USA

- This paper describes a software package, genome-sampler, that subsamples collections of SARS-CoV-2 genomes with attention to various metadata attributes. The paper is well motivated and well written.
- My review focuses on the tutorial and getting the software running.
- I was pleased to see that the authors posted their source code to an archival location (Zenodo).
- I would encourage the authors to specify a release (rather than the latest branch) to install in the tutorial. Similarly, the conda install instructions in the tutorial should pin the versions of the software; this could easily be done via an environment yml provided within the genome-sampler github. And (minor nit) specifying '-y' in the copy/paste command line for conda install would be good too!
- The tutorial downloads FASTA data from a Dropbox. This is brittle and should be changed. I suggest using an archive (Zenodo, osf.io, DataDryad) for this data, and making it copy/paste downloadable. They're small enough to put in github and version, too.
- The tutorial downloads spreadsheet data from a Google Docs spreadsheet, and I would encourage the authors to put this data in git as well - even if there's a pedagogical reason to ask users to go through downloading from a spreadsheet, it's brittle to have a content-mutable and unversioned URL be the only location for the tutorial data.
- The approach of downloading the Snakefile is also brittle. I suggest revamping the tutorial so that it does a local install of a git clone; see e.g. this documentation (for an alpha package) that would be a simpler approach, I think:
  https://github.com/dib-lab/charcoal/tree/51caa9f034f3d301367cb6eea2ee96b5e1ea05bb#quickstart
- Note that snakemake supports config files that let you put the overridable configuration parameters in a YAML file. This might be nicer than editing the Snakefile directly. Happy to provide detailed examples on request.
- While I'm suggesting things, you could also use conda environments in the Snakefile to obviate the qiime etc. install. Then you'd just need to install snakemake and run it with -- use-conda to get it all done.
- When running the tutorial, I see the following error:
  ...
  /qiime2/sdk/util.py", line 92, in parse_format
  raise TypeError("No format: %s" % format_str)
  TypeError: No format: GISAIDDNAFASTAFormat
  I'm not sure where to go from here. I've filed an issue with the full details.

Are the conclusions about the tool and its performance adequately supported by the findings presented in the article?

Yes

Is the rationale for developing the new software tool clearly explained?

Yes

Is the description of the software tool technically sound?

Partly

Are sufficient details of the code, methods and analysis (if applicable) provided to allow replication of the software development and its use by others?

Partly

Is sufficient information provided to allow interpretation of the expected output datasets and any results generated using the tool?

Yes

Reviewer Expertise:

bioinformatics and software development

I confirm that I have read this submission and believe that I have an appropriate level of expertise to confirm that it is of an acceptable scientific standard, however I have significant reservations, as outlined above.

# Response

**Published:** 2020-08-27

**Authors:** C. Titus Brown

**Affiliations:** University of California, Davis, USA

I have managed to run the pipeline successfully, and have the following additional comments --

 ---

 I successfully ran the tutorial, huzzah!

 It would be good to add a rough estimate of resources required (memory,

 disk, CPU time) to the tutorial.

# Response

**Published:** 2020-10-17

**Authors:** Greg Caporaso

**Affiliations:** Northern Arizona University, USA

Thank you for the feedback on our manuscript, and for testing the software and offering suggestions. Below we provide a point-by-point reply. Your comments are presented in italics, and we reply to each in-line. 

*This paper describes a software package, genome-sampler, that subsamples collections of SARS-CoV-2 genomes with attention to various metadata attributes. The paper is well motivated and well written.*

**

*My review focuses on the tutorial and getting the software running.*

**

*I was pleased to see that the authors posted their source code to an archival location (Zenodo).*

**

*I would encourage the authors to specify a release (rather than the latest branch) to install in the tutorial. Similarly, the conda install instructions in the tutorial should pin the versions of the software; this could easily be done via an environment yml provided within the genome-sampler github. And (minor nit) specifying '-y' in the copy/paste command line for conda install would be good too!*

We agree with the reviewer’s suggestion and have updated our install instructions to install from a specific release. The conda installation now includes specific pinned versions of dependencies. (We prefer to not include the -y option, but rather have the user acknowledge that changes are about to be made to their system, so we have not included that.)

*The tutorial downloads FASTA data from a Dropbox. This is brittle and should be changed. I suggest using an archive (Zenodo, osf.io, DataDryad) for this data, and making it copy/paste downloadable. They're small enough to put in github and version, too.*

We also agree with this suggestion. As of our most recent release, the tutorial files are now packaged in the GitHub repository and are included in the updated Zenodo package that we have prepared for the most recent release and paper resubmission. 

*The tutorial downloads spreadsheet data from a Google Docs spreadsheet, and I would encourage the authors to put this data in git as well - even if there's a pedagogical reason to ask users to go through downloading from a spreadsheet, it's brittle to have a content-mutable and unversioned URL be the only location for the tutorial data.*

As of our most recent release, these files are now packaged in the GitHub repository and are included in the updated Zenodo package.  

*The approach of downloading the Snakefile is also brittle. I suggest revamping the tutorial so that it does a local install of a git clone; see e.g. this documentation (for an alpha package) that would be a simpler approach, I think:*

*https://github.com/dib-lab/charcoal/tree/51caa9f034f3d301367cb6eea2ee96b5e1ea05bb#quickstart*

We have updated the tutorial so that this file is downloaded from a release version of genome-sampler, which is less brittle. We prefer to not have users install from a git clone directly, since we are officially supporting conda installation (which tends to be easier for our novice user), and adding a second officially supported installation method would add to our technical support burden. 

*Note that snakemake supports config files that let you put the overridable configuration parameters in a YAML file. This might be nicer than editing the Snakefile directly. Happy to provide detailed examples on request.*

We thank the reviewer for this suggestion. We now use a Snakemake config file to configure these parameters.

*While I'm suggesting things, you could also use conda environments in the Snakefile to obviate the qiime etc. install. Then you'd just need to install snakemake and run it with -- use-conda to get it all done.*

This is an attractive option and we invested time in exploring this approach. We found that our platform-specific environment files complicate this, so we ultimately opted to not take this suggestion. Specifically, users would need to replace the environment path for every rule in the Snakefile (if they were using OS X instead of Linux, for example). We have simplified our installation workflow (both for Snakemake users and non-Snakemake users) and hope that our provided workflow can serve as a template for more sophisticated use-cases, such as rule-specific conda or singularity containers.

*When running the tutorial, I see the following error:*

*...*

*/qiime2/sdk/util.py", line 92, in parse_format*

*raise TypeError("No format: %s" % format_str)*

*TypeError: No format: GISAIDDNAFASTAFormat*

*I'm not sure where to go from here. I've filed an issue with the full details.*

We haven’t come across this error, but it is likely related to genome-sampler not being installed correctly. Our updated install instructions should simplify this. We’re happy to try to help work out the problem, but we haven’t seen this issue on our issue tracker. We have not had reports of other users running into this (but we have had other support requests, so we know that this is not something that is preventing people from using genome-sampler).

# Peer review: Reviewer response for version 1

**DOI:** 10.5256/f1000research.27305.r65756

**Published:** 2020-07-31

**Authors:** James Hadfield

**Affiliations:** 1 Vaccine and Infectious Disease Division, Fred Hutchinson Cancer Research Center, Seattle, WA, USA

This paper presents a software tool to tackle a pressing, but welcome, problem: the number of publicly shared SARS-CoV-2 sequences (c. 75,000 at the time of this review) are too numerous to be analysed or visualised using currently available methods in a time-frame relevant for understanding local outbreaks. There is a need for researchers to be able to interrogate a particular collection of samples in a wider (often worldwide) context, and the choice of such context will greatly influence the conclusions drawn. The approach presented here samples contextual sequences by considering temporal, geographical and sequence-similarity data.

 As the paper notes, similar approaches are available and in use by the wider community, however there is value in creating a range of tools for researchers to employ as required and which best suit their needs. This tool is easily installable and provides a ready-to-use solution for a pressing problem. It is purposefully designed in such a way that it is interoperable with other approaches, and will be immediately useful to researchers.

 I agree with the authors that a thorough evaluation and comparison of different subsampling approaches is beyond the scope of this paper, however there are some aspects which were not discussed in the manuscript which should be addressed (i.e. minor revisions required). Please note that this review focuses on the genome-sampler software described here, and is not a commentary on the wider QIIME 2 platform.

**Installation**

 Following https://caporasolab.us/genome-sampler/tutorial.html, installation was straightforward and the provided example worked out of the box. The tutorial was well written and didn't require any background knowledge of QIIME 2. The authors should be commended for this.

**Points to address**

1. Time required. The example data provided (10 focal, 75 contextual sequences) took c. 40 minutes running on a laptop using a single core. As this tool will commonly be employed using all publicly available data as context (currently c. 75,000 genomes) an overview of the time (as well as memory & parallelizability) required to perform subsampling for various numbers of focal & contextual sequences should be provided.
2. Aligned genomes are not required as input as vsearch is used to compare genomes. My understanding is that vsearch will perform (a relatively fixed number of) pairwise alignments for each focal genome vs. the contextual data set to gauge percent identity. The paper would benefit from a short explanation of why this approach was used rather than aligning all sequences (e.g. to a reference genome).
3. There is no ability to subsample focal sequences. As the authors correctly mention in the introduction, the impressive rate of genome sequencing presents a number of challenges. It is already a reality that certain locally-derived (focal) datasets are large enough to require subsampling of their own, and this will become more common over time. The authors should address this by either implementing the ability to sample focal sequences or prescribing that the researcher must define a suitably small focal set.
4. The rapid submission of samples to public repositories  should to be facilitated as much as possible. It may be commonplace to have focal samples which are also present in the contextual data. Could the authors detail what would happen in this case (e.g. are the contextual "duplicates" removed or will they bias steps 3 & 4 as they may preclude the inclusion of other samples?), or does this use-case need to be avoided by the user?

**Minor points**

- Step 5 is not annotated on figure 1.
- [page 3, paragraph 3] Sampling across time won't necessarily allow the reliable inference of a clock signal, but is rather a prerequisite.
- [page 4, step 1(iii)] Are short sequences (i.e. those with lots of gaps) excluded here, or only those with a large proportion of Ns?
- [page 3, step 3] Reword to clarify that this step is performed per-sample, i.e. that the (10) closest matches are found for each sample in the focal set.

Are the conclusions about the tool and its performance adequately supported by the findings presented in the article?

Yes

Is the rationale for developing the new software tool clearly explained?

Yes

Is the description of the software tool technically sound?

Yes

Are sufficient details of the code, methods and analysis (if applicable) provided to allow replication of the software development and its use by others?

Yes

Is sufficient information provided to allow interpretation of the expected output datasets and any results generated using the tool?

Yes

Reviewer Expertise:

Bioinformatics, phylogenetics

I confirm that I have read this submission and believe that I have an appropriate level of expertise to confirm that it is of an acceptable scientific standard.

# Response

**Published:** 2020-10-16

**Authors:** Greg Caporaso

**Affiliations:** Northern Arizona University, USA

Thanks for your thoughtful review of our manuscript! We have reviewed your comments and are submitting a revision that addresses them as detailed here. The reviewer comments are presented in italics, and our replies to each follow in-line. 

*Installation*

*Following https://caporasolab.us/genome-sampler/tutorial.html, installation was straightforward and the provided example worked out of the box. The tutorial was well written and didn't require any background knowledge of QIIME 2. The authors should be commended for this.*

Thank you! 

*Points to address*

*Time required. The example data provided (10 focal, 75 contextual sequences) took c. 40 minutes running on a laptop using a single core. As this tool will commonly be employed using all publicly available data as context (currently c. 75,000 genomes) an overview of the time (as well as memory & parallelizability) required to perform subsampling for various numbers of focal & contextual sequences should be provided.*

In response to this request we have added a new figure to this paper presenting a benchmark of CPU time and memory usage for different sized context and focal genome data collections. Our results are presented in Figure 1.  

*Aligned genomes are not required as input as vsearch is used to compare genomes. My understanding is that vsearch will perform (a relatively fixed number of) pairwise alignments for each focal genome vs. the contextual data set to gauge percent identity. The paper would benefit from a short explanation of why this approach was used rather than aligning all sequences (e.g. to a reference genome).*

In the ideal scenario, for our sample_diversity step, we would align all genomes in a collection against all other genomes in that collection, compute all similarities between genomes, and then use those pairwise similarities to cluster genomes based on their similarity. We could then select one or more sequences from each cluster for downstream analysis, which would ensure that we have represented the diversity of the genome collection. Computing all pairwise alignments is too time consuming for most practical applications however, so we use vsearch’s heuristic approach which tries to achieve the same goal but by reducing the number of pairwise alignments that are computed by trying to prioritize alignments between each sequence and sequences that are suspected to be highly similar to it. This provides higher resolution than the all-against-one approach that the reviewer mentions, because for example, if two query sequences (say ACGTT and AGGTA) are aligned to a single reference sequence (say ACGTA), we know that both are 80% similar to the reference, but we don’t know how similar those sequences are to each other (they could be 60% similar to each or 100% similar to each other in this example). Thus we could represent distance to a reference sequence reasonably well using the all-against-one comparison, but we wouldn’t know if we had sampled the full diversity represented in the genome collection. Since we were interested in using the vsearch approach for the sample_diversity step, it made sense to us to also use this for the other steps in the workflow. 

*There is no ability to subsample focal sequences. As the authors correctly mention in the introduction, the impressive rate of genome sequencing presents a number of challenges. It is already a reality that certain locally-derived (focal) datasets are large enough to require subsampling of their own, and this will become more common over time. The authors should address this by either implementing the ability to sample focal sequences or prescribing that the researcher must define a suitably small focal set.*

It actually is possible to sample the focal sequences using the same approaches that are applied to the context sequences in our tutorial, though we acknowledge that that was not clear (and undocumented) in our original submission. In the revised documentation included with our updated release we have added a section that specifically discusses this point. See the new section, Sampling focal sequences.  

*The rapid submission of samples to public repositories should to be facilitated as much as possible. It may be commonplace to have focal samples which are also present in the contextual data. Could the authors detail what would happen in this case (e.g. are the contextual "duplicates" removed or will they bias steps 3 & 4 as they may preclude the inclusion of other samples?), or does this use-case need to be avoided by the user?*

This is a great point which we did not address in our initial version of the documentation. This situation would bias the sample-neighbors step in particular, as those duplicated sequences would be very likely to be chosen as the nearest neighbors. We have added a note to the main tutorial, and a new section to the documentation Removing sequences present in both focal and context sequence collections, illustrating how the user can address this. 

*Minor points*

*Step 5 is not annotated on figure 1.*

Thanks for pointing out this omission. We have updated the figure to fix this. 

*[page 3, paragraph 3] Sampling across time won't necessarily allow the reliable inference of a clock signal, but is rather a prerequisite.*

We modified the text the reviewer is referring to clarify this point. 

*[page 4, step 1(iii)] Are short sequences (i.e. those with lots of gaps) excluded here, or only those with a large proportion of Ns?*

Since the input sequences for genome-sampler are unaligned, any gap characters (- or .) are stripped from the sequences on import. At this step of the analysis, the user does have the ability to filter sequences based on their length (sequences with length less than a minimum length or greater than a maximum length, both of which can be specified by the user, are optionally filtered). This has been clarified in the text. 

*[page 3, step 3] Reword to clarify that this step is performed per-sample, i.e. that the (10) closest matches are found for each sample in the focal set.*

We have modified the text to clarify this.
